# A Few Considerations in Filling Teeth

**Published:** 1899-05

**Authors:** C. N. Johnson

**Affiliations:** Chicago, Ill.


AMERICAN JOURNAL

—OF—

DENTAL SCIENCE.

VOL. XXXIII. BALTIMORE, MAY, 1899. NO. I.

### Selections.

ARTICLE I.

### A FEW CONSIDERATIONS IN FILLING TEETH.

C. N. JOHNSON, L. D. S., D. D. S., CHICAGO, ILL.

### APPROXIMAL CAVITIES IN BICUSPIDS AND MOLARS.

The principles involved in the treatment of caries occurring on the approximal surfaces of bicuspids are so similar to those occurring on like surfaces of molars that they will be considered as one class of cavities. Minor differences in the detail of the work will, it is true, be called for, but these are readily suggested by the differences in the forms of the teeth. The position and function of bicuspids and molars are nearly identical, and they are subject to practically the same influences leading to decay primarily and to a recurrence of decay around fillings. The same forces are at work to dislodge fillings, and the same general plan of anchorage must be pursued in the one as in the other. For these reasons they are treated in common.

SIMPLE APPROXIMAL CAVITIES NOT INVOLVING OTHER SURFACES.

The instances are very rare where it may be deemed advisiable to fill this kind of a cavity. Usually when decay begins on the approximal surface of a bicuspid or molar, the proper preparation of the cavity involves its extension through to the occlusal surface. Almost the only exceptions relate to those cases where the cavity faces an open space caused by the loss of a tooth, or to those occasional instances where there has been extensive recession of the gums in the interproximal space and consequent decay in the cervical region. This latter usually occurs in advanced age, when such extensive cutting as would be necessary to involve the occlusal surface would not be justifiable, and where the open interproximal space admits of access from the buccal aspect. The farther rootwise the decay occurs the stronger the argument for filling without extending occlusally, on account of the better facility for approach and the greater thickness of the occlusal wall.

but in ordinary caries occurring near the contact point, and with the teeth standing in line one against the other, the rule should be to open the cavity to the occlusal surface. The reasons for this lie in the fact that in such cases access cannot be gained to do perfect work short of very extensive separation, and then when the teeth have been so separated and filled, and have fallen back to their original position, an element of danger to the filling remains on account of the margin of the filling being too near the contact point. The reason that decay begins in this locality in the first instance is because a certain area of the tooth-substance is left exposed to the action of micro-organisms, undisturbed by friction of the tongue, cheeks, the tooth-brush, or of food in the process of mastication. If the line between enamel and filling be left near the contact point, the same influences which induced the original decay may be expected to act on the enamel at the margin of the filling to bring about a recurrence. If the cavity is extended occlusally far enough to make a clean margin, the occlusal wall is thereby rendered too weak to withstand mastication. All operations performed upon these surfaces without extension must therefore be considered in the light of temporary work.

Some times such fillings do good service through the care with whidh they are inserted, coupled with the probable fact that just at this time the patient acquires a partial or complete immunity from caries. Many indifferent operations have received credit for being perfect owing to this very fact of immunity, and the whole question of periodical immunity and susceptibility of our patients in regard to the attack or progress of dental caries should receive more careful consideration than it does. But what concerns us now in treating the present subject is that clinical experience proves most of these small fillings to be temporary, and resort should be had to their occasional insertion with this fact clearly in mind.

The plan of anchorage for such fillings is very simple. There is little stress to dislodge the filling, and all that seems necessary in the formation of the cavity is to make parallel walls surrounding it at right angles with the axial wall, and to bevel the enamel margins.

PROXIMO-OCCLUSAL CAVITIES IN BICUSPIDS AND MOLARS.

Wherever decay has so invaded the approximal surface as to involve the occlusal surface, or wherever it is deemed necessary to open the cavity to the occlusal surface in those cases having this wall still remaining, a new class of conditions confront the operator. In view of the fact, as already intimated, that most approximal cavities in these teeth must be made to include the occlusal surface, it becomes necessary to study somewhat carefully the conditions governing the treatment of such cases.

The principal objects to be attained in the insertion of this kind of a filling are, first, to check the existing decay; second, to prevent, so far as possible, a recurrence of decay in the future; third, to securely anchor the filling against displacement from the stress of mastication; and, fourth, to so restore the original form of the tooth that it will be maintained in its proper relation with the other teeth and with the gum-tissue filling the interproximal space.

The first of these requirements may be met by simply removing the decay and inserting a filling with perfect margins; and this would seem to be the limit of attainment with many operators. A failure to recognize other necessities in the case is accountable for much of the disappointment following these operations, through the temporary nature of this kind of work.

The same general rules of extension for prevention applying to these cavites that were given for approximal cavities in the anterior teeth, except that in bicuspids and molars esthetic considerations do not so materially affect the case, and the rules may therefore be less frequently waived on this account. The usual points of recurrence of decay around these fillings are at the cervico-buccal and cervico-lingual angles of the cavity, though in cavities left very narrow bucco-lingually the entire buccal and lingual margins may become involved.

Another form of failure, where cavities are narrow in the region of the marginal ridge, relates to a fracture of the enamel at the proximo-occlusal angles. Enamel left in this form is easily broken down by the stress of mastication, causing a break between the filling and the margin.

The plan of anchorage for these fillings calls for careful consideration along the lines of the most approved mechanical principles, and with a due regard for the location of the filling and the probable stress to which it will be subjected in the process of mastication. The force of mastication varies greatly in different individuals, and the intelligent operator will take cognizance of this and govern his operations thereby.

In estimating the probable durability of a filling and the extent of anchorage required to maintain it in place, a careful study should be made of the markings of mastication in the mouth under treatment. The expression "markings of mastication" is coined for the purpose of directing attention to this form of study. Mastication leaves its marks plainly and indelibly upon the teeth and upon fillings placed in them, and these markings offer a good index for the observant operator to estimate the probable average force exerted in ordinary mastication in a given mouth. The use of the gnathodynamometer for the purpose of recording the stress of mastication, while very valuable for scientific study and for throwing much light on the possible force of mastication, is not considered to be the most reliable index to the force actually employed in the comminution of food. The greatest possible force that can be exerted in closing the jaws is often far removed from the actual force used in mastication in the same mouth, and is not invariably relative to it. For this reason, if we accept it as our sole guide for the extent of anchorage required for our fillings, we shall in some instances subject our patients to unnecessarily broad and painful cutting to accomplish an object which might have been attained by less heroic means. On the other hand, we may sometimes fall short of adequate anchorage in those cases where the gnathodynamometer gives a low record, but where the actual wear and tear on fillings in mastication is somewhat severe.

It is true we should aim in all cases to anchorage our fillings in such security that the greatest possible stress of which the jaws are capable will not dislodge them, but the conditions under which we are compelled to operate will not invariably permit it. The requisite bulk of tooth tissue is not always left for us by decay, and the sensibilities of our patient must also be considered. The same statement is true here which was used in connection with cutting cavities in anterior teeth, that we must jeopardize the nervous system of our patient to follow out some heroic theory. The fact of ignoring the patient in these matters by a blind pursuit of an ideal in the mind of the operator is accountable for much of the aversion experienced against the dental chair, and we must have a care not to discourage people against permitting dental service to be done for them by too great a degree of severity during the operation. This does not imply that we must be slipshod in our methods, or that we must at all times avoid giving pain. It is occasionally necessary to give pain, but the operator should carefully study his patient and limit the discomfort to a reasonable degree of tolerance. (This matter will be considered more in detail in a subsequent chapter on the treatment of sensitive dentin).

The markings of mastication relate to worn surfaces of enamel at points of occlusal contact which are evidently formed by mechanical wear instead of by erosion, to deep indentations in fillings made by repeated and vigorous thrusts of the opposing cusp, and occasionally to fractured and jagged enamel showing evidence of rough usage. If the operator will give a careful study to the condition of his patients' teeth and watch for these markings he will soon be able to tell quite accurately the probable degree of service which a given set of teeth are called upon to do at table, and it will often guide him in his methods of anchoring fillings.

The different plans of anchorage for these proximo-occlusal fillings in bicuspids and molars deserve careful consideration. The method almost universally employed in the past has been to cut a groove along the buccal and lingual walls of the cavity, and in some instances to groove the cervical wall. The limitations of this method relate to the insecurity of such anchorage against heavy stress, and to the danger of weakening the walls, particularly in those cases where the buccal and lingual outlines of the cavity are sufficiently extended for safety against recurrence of decay. Unless the grooves are very broad and deep—a condition disastrous to cavity walls—any tipping stress on such a filling will tend to lift it slightly away from the axial wall, leaving a leak along the filling at that point. Then, again, the occlusal aspect of this form of filling is ordinarily unsatisfactory. In bicuspids especially the filling encounters a fissure running mesio-distally between the cusps, and leaving at the junction of the filling and fissure a shoulder on the filling impossible of perfect finish, and an element of weakness in the probable recurrence of decay along the fissure. A fissure is invariably a defect in the tooth whereby two islands of calcification have failed to coalesce in its development, leaving a break in the continuity of the enamel at that point and a crevice for the ingress of deleterious matter. It should therefore be the constant rule that whenever a cavity on the occlusal surface of a tooth encounters a fissue, the fissue should be drilled out to its extreme end and included in the cavity.

What would appear to be a much preferable method of anchorage to that just considered, and one offering greater security to the filling, is to create a step on the occlusal surface of the tooth at right angles with the approximal portion of the cavity, and extending sufficiently into the occlusal surface to effectively lock the filling into place. This also results in the obliteration of the fissure and the formation of a filling easy of finish.

With this form of anchorage the filling can not be displaced short of a fracture of the filling or a stretching of the material at the point where the approximal portion joins the occlusal through repeated impacts of the opposing cusp in mastication. To prevent this the cavity should be made deep enough at this point to allow of sufficient bulk of filling material for strength, and the material should be thoroughly packed and well condensed to give it the greatest degree of resisting power.

Another point in connection with the dislodgment of these fillings relates to the form of the filling on the occlusal surface, and also the form of the cusps on the opposing teeth. While the general rule holds good that in the formation of fillings they should be made as nearly as possible to reproduce the original form of the tooth, yet in these proximo-occlusal fillings a slight modification of the original form is often advisable. In the national form we find the marginal ridge of enamel standing more prominent than the enamel between the cusps, thereby receiving greater impact in the process of mastication. If in the insertion of the filling we reproduce the marginal ridge, we subject the filling to too great leverage at a point where it has a tendency to tip it away from its anchorages. This may be avoided by making the filling as high as possible midway between the cusps, and sloping it toward the contact point in such a way as to avoid making a marginal ridge and to present a gradual incline from the highest point between the cusps to the point of contact on the approximal surface. In doing this the occlusal surface of the filling is so presented to the cusp of the opposing tooth that the tendency on closure of the jaws is to force the filling laterally into the cavity instead of lifting it away, as would result if the marginal ridge were reproduced.

This plan does not materially impair the efficiency of the tooth for mastication, but even if it did lessen the masticating area somewhat it would still be justifiable on the ground that a tooth with its masticating area reduced one-half, but containing a filling safely anchored and enamel so sloped as to avoid fracture, is more valuable than one presenting a full masticating area subject to the danger of filling displacement and fractured enamel-walls. This does not imply that the filling should be made narrower mesio-distally at the contact point than the tooth originally was. For reasons which will appear later the full width of the tooth must be maintain wherever possible. This may result in some instances in the point of contact with the approximating tooth being carried slightly rootwise of the original contact, but if care is exercised this may safely be done without impairing the efficiency of the contact or inerferring with the gum-tissue in the interproximal space.

The treatment of the cusps of opposing teeth coming against these fillings is a matter of much importance, particularly with bicuspids. When the sharp buccal cusp of a lower bicuspid impinges so far into a cavity on the opposing upper bicuspid as to necessitate making the filling too thin for strength, or where it passes so far between the cusps of the upper tooth as to form a wedge capable of splitting the tooth, the tip of the cusp on the lower bicuspid should be ground down so as to shorten it and present a broad surface to the upper tooth instead of a wedge shape. This will result in the formation of a thicker and stronger filling in the decayed tooth, and such a change in the direction of force exerted by the lower tooth as to minimize the danger of spliting its opponent. With a wedge-shaped cusp there is much lateral force exerted buccally and lingually against the cusps of the upper tooth, but with a broad, flattened cusp the direction of force is more nearly parallel with the long axis of the tooth and the tendency to split is lessened. This grinding, if done judiciouly, will not interfere with the usefulness nor impair the integrity of the lower bicuspid. The enamel is very thick at that point, and there is little liability to decay, so that this method should be employed quite frequently for the greater permanence of our fillings and the greater safety of the teeth, especially in those cases where the cusps of the lower teeth are very prominent and sharp.

One of the most important considerations in the management of these proximo-occlusal cavities relates to the form of the filling on the approximal surface. It should be so built out to a contour that the tooth will be maintained in its proper position in the arch, and that the gum-tissue in the interproximal space shall be protected and preserved in a healthy condition. When the teeth stand in their normal relation in the jaws they are supported on their approximal surfaces by contact with the tooth next in line, and the interproximal space between these points of contact and the border of the alveolar process is filled with gum-tissue. This gum-tissue has an arched form buccolingually, with the crest of the arch near the contact point; and this form facilitates the cleansing of the space by a deflection of the food buccally and lingually in mastication. So long as the contact points are small and the space of normal form and filled with gum-tissue, foreign material will not find a lodgement in the space. In the comminution of fibrous food, such as meat, the fibers may occasionally be forced between the contact points, but they are not retained there on account of the narrowness of contact. The succeeding excursion of food on closure of the jaws in passing buccally and lingually along the incline of gum will catch them and carry them with it, leaving the space clean.

When decay takes place on the approximal surface and the contact point breaks down, the teeth, lacking approximal support, have a tendency to drop together, forcing the gum from between them and narrowing the space. In cases of extensive caries the teeth may so change their position as to practically obliterate the space and crush out all of the gum tissue, leaving the buccal and lingual festoons of the gum more prominent than that portion midway between the teeth. This results in an inverted arch to the gum, and produces a pocket between the teeth which is especially favorable to the reception and retention of food debris. When caries occurs in this way the necessary procedure to restore the gum to health is to wedge the teeth apart to their original position, and then to so contour the filling that they will be maintained there.

If the filling is inserted without this precaution the result is a broad, flat approximal surface to the filling, which will catch fibers of food and retain them to decompose. This wedging of food between teeth is an element of great discomfort to the patient, and a prolific source of failure in these approximal fillings. It not only results in recurrence of decay, but sadly impairs the health of the gum and pericemental membrane. No operation should be considered satisfactory which does not include in its performance a due regard for the form of the interproximal space and the health of the gum-tissue within it.

The attempt to prevent food from wedging between teeth by making broad contacts built tightly against the approximating tooth usually fails in its object through the fact that contact can not in this way be made so perfect that at times the individual movement of the teeth one against the other will not result in the passage of fibrous food between them. When it once makes its way between the filling and the approximating tooth, it is firmly held there by the broad contact. The only safe form to give these fillings is to make a narrow rounded contact, sufficiently dense to maintain the teeth in position and to preserve its form against the wear occasioned by the individual movement of the teeth. This wear is sometimes quite severe, as is shown by the facets worn in the enamel on the approximal surfaces of many sound teeth. This recalls the fact that these worn facets are often a prolific source of discomfort to the patient, even where there is no decay; and in those cases where a filling is being built in a cavity approximating a tooth with an extensive facet, the margins of the facet should be slightly rounded off to produce an oval form to the surface. The attention of the operator should not be limited to the single tooth being filled, but he should study carefully the adjacent parts, to the end that the teeth and gums in the entire region be placed in the best possible condition.—*Dental Cosmos.*

